# Associations between inflammatory markers, body composition, and physical function: the Copenhagen Sarcopenia Study

**DOI:** 10.1002/jcsm.12832

**Published:** 2021-10-27

**Authors:** Rikke S. Kamper, Julian Alcazar, Lars L. Andersen, Bryan Haddock, Niklas Rye Jørgensen, Peter Hovind, Charlotte Suetta

**Affiliations:** ^1^ Geriatric Research Unit, Department of Geriatric and Palliative Medicine Copenhagen University Hospital, Bispebjerg and Frederiksberg Copenhagen Denmark; ^2^ Department of Clinical Physiology, Nuclear Medicine and PET Rigshospitalet Glostrup Glostrup Denmark; ^3^ GENUD Toledo Research Group Universidad de Castilla‐La Mancha Toledo Spain; ^4^ Geriatric Research Unit, Department of Medicine Copenhagen University Hospital, Herlev and Gentofte Herlev Denmark; ^5^ The National Research Centre for the Working Environment (NFA) Copenhagen Denmark; ^6^ CopenAge—Copenhagen Center for Clinical Age Research University of Copenhagen Copenhagen Denmark; ^7^ Department of Clinical Biochemistry Rigshospitalet Glostrup Denmark; ^8^ Department of Clinical Medicine, Faculty of Health and Medical Sciences University of Copenhagen Copenhagen Denmark; ^9^ CIBER of Frailty and Healthy Aging (CIBERFES) Madrid Spain; ^10^ Department of Clinical Physiology and Nuclear Medicine University Hospital, Bispebjerg and Frederiksberg Copenhagen Denmark

**Keywords:** Inflammation, Ageing, Muscle mass, Muscle strength, Physical function

## Abstract

**Background:**

Chronic low‐grade inflammation has been suggested as one of the key elements in the development of sarcopenia, but in contrast to disease‐related loss of muscle mass, the role of chronic low‐grade inflammation in age‐related (primary) sarcopenia is still not clear. The aim of this study was to investigate low‐grade inflammation in relation to age and the potential association between inflammatory biomarkers and body composition, muscle strength and physical performance in a healthy Danish cohort.

**Methods:**

There were 1160 generally healthy men and women (range: 22–93 years) included. Appendicular lean mass (ALM) and visceral fat normalized to height (kg/m^2^) was assessed by dual‐energy X‐ray absorptiometry (iDXA, GE Lunar). Muscle strength and physical performance were evaluated by handgrip strength (HGS), 30 s sit‐to‐stand performance, and maximal gait speed (GS). Systemic levels of TNF‐α, IL‐6, IL‐1β, IL‐4, IL‐13, and IFN‐γ were measured using multiplex bead‐based immunoassays (Bio‐Rad). hsCRP was assessed using latex particle‐enhanced immunoturbidimetric assays (Roche Diagnostics).

**Results:**

With age, ALM/h^2^, HGS, sit‐to‐stand performance and GS decreased, whereas visceral fat/h^2^ increased in both men and women (*P* < 0.05). Systemic levels of hsCRP, TNF‐α, IL‐4, and IFN‐γ increased with age in men and women (*P* < 0.05), while IL‐1β increased in women only (*P* < 0.01). Higher levels of hsCRP were associated with lower ALM/h^2^ in elderly (≥65 years) men and women (*P* < 0.001). Higher levels of hsCRP were associated with lower handgrip strength in elderly women (*P* < 0.05) whereas higher levels of hsCRP was not associated with lower HGS in elderly men (*P* = 0.056). Higher levels of hsCRP were associated with lower GS (*P* < 0.05), whereas IFN‐γ was positively associated with GS in elderly women (*P* < 0.05), but not elderly men. Visceral fat index was positively associated with hsCRP in elderly men and women (*P* < 0.001). Compared with elderly with normal HGS, elderly men and women with low HGS displayed higher levels of TNF‐α and hsCRP (*P* < 0.05).

**Conclusions:**

With age, systemic levels of hsCRP, TNF‐α, IL‐4, and IFN‐γ increased, with hsCRP and TNF‐α being especially elevated in more physically frail elderly supporting the association between low‐grade systemic inflammation and poor physical function. In contrast, only high levels of hsCRP were weakly associated with low muscle mass and positively associated with visceral fat and low physical function, suggesting that chronic low‐grade inflammation is not the main driver of age‐related loss of muscle mass as previously suggested.

## Introduction

Ageing is a multifaceted biological process with important hallmarks such as changes in body composition, sarcopenia, and chronic low‐grade inflammation.^1^, ^2^, ^3^ The term “inflammaging” was introduced by Franceschi and colleagues at the beginning of the new millennium to characterize the chronic two‐ to fourfold increase in circulating levels of inflammatory biomarkers typically observed with ageing.^2^, ^4^ Chronic low‐grade inflammation and sarcopenia have independently been associated with adverse health outcomes, such as disability, loss of independence, morbidity, mortality, and hospitalization.^5^, ^6^

The aetiology of sarcopenia is still not fully elucidated but is believed to be a multifaceted interplay between physical inactivity, changes in the neuromuscular system, lower levels of anabolic hormones as well as chronic low‐grade inflammation.^7^, ^8^, ^9^, ^10^, ^11^ But, whereas high cytokine levels in acute or chronic disease states are known to accelerate the development of sarcopenia,^12^, ^13^ the role of chronic low‐grade inflammation for loss of muscle mass in otherwise healthy older individuals (primary sarcopenia) is less clear.^12^ The relationship between inflammatory biomarkers and muscle mass, muscle strength, and physical function has traditionally been investigated by assessing a single or limited number of inflammatory proteins^14^ and although CRP, IL‐6 and TNF‐α are among the most investigated inflammatory markers, no single biomarker has yet been consistently associated with the negative physical outcomes observed with ageing.^12^, ^14^

A recent systematic review and meta‐analysis on markers of inflammation and their association with muscle mass and muscle strength concluded that higher systemic inflammation was associated with lower muscle strength and muscle mass, but also questioned the temporal relationship between inflammatory markers and measures of muscle mass or strength owing to a high frequency of cross‐sectional designs and inconsistent or weak longitudinal associations. Hence, the authors argued the need for clearly defined study populations.^12^ The aim of this study was therefore to investigate a range of inflammatory biomarker profiles in a large healthy cohort aged 22–93 years and further to examine the potential association between inflammatory biomarkers and muscle mass, visceral fat, muscle strength, and physical function in healthy older well‐functioning individuals.

## Methods

### Study cohort

The current study uses data from the Copenhagen Sarcopenia Study, a population‐based cross‐sectional study conducted at Copenhagen University Hospital Rigshospitalet, Glostrup, from December 2013 to June 2016. More detailed information regarding The Copenhagen Sarcopenia study has been described elsewhere.^15^ In brief, the study included men and women aged 20 to 93 years, living in the Copenhagen metropolitan area, who were all characterized as living independently and being apparently healthy. Exclusion criteria were pregnancy, acute or chronic medical illness, cancer, surgery within the last 3 months, use of corticosteroids, and any history of compromised ambulation or prolonged immobilization. For the present study, participants lacking inflammatory data (*n* = 70), body composition (*n* = 11) or data for strength or physical function (*n* = 12) were excluded, leaving data on 1160 participants (men: *n* = 521, mean age = 58.2 ± 16.7 years; women: *n* = 639, mean age = 58.7 ± 17.9 years) for analyses. Written, informed consent was obtained from all participants, and all investigations were conducted in accordance with the Declaration of Helsinki II and approved by the Ethical Committee of Copenhagen (H‐3‐2013‐124).

### Anthropometric measurements and body composition

Height (cm) was assessed without shoes to the nearest 0.1 cm. Weight (kg) was measured to the nearest 0.1 kg without shoes, wearing light clothing. Whole body dual‐energy X‐ray absorptiometry DXA exams were acquired as per the manufacturer's instructions on an iDXA fan beam densitometer (GE Lunar, Madison, WI, USA). The same scanner was used throughout the study, and whole‐body scans were carried out by trained technicians. Analyses were performed using Encore software version 16.0. Appendicular lean mass (ALM) was defined as the sum of lean soft tissue from the arms and legs and relative ALM was acquired by normalizing ALM to height^2^ (kg/m^2^) to account for body size. Visceral fat index was acquired by normalizing VF to height^2^ (kg/m^2^) to account for body size.

### Assessment of muscle strength and physical function

In all participants, handgrip strength (kg) was assessed using a handheld dynamometer (Jamar, Sammons Preston Rolyan, Chicago, Illinois, USA) with participants in a seated upright position with the elbow flexed at 90° and the arm supported by a horizontal surface. All participants had three successive maximum force trials with each hand, as described in detail elsewhere.^15^ The maximum value was used as the final HGS score.

The 30 s chair stand test was used to assess sit‐to‐stand performance (STS) as the number of times a participant was able to rise and sit from a standardized chair (no armrest, height 45 cm) in 30 s. The participant was seated with the back straight and the arms folded across the chest. Following a verbal cue, the participant was instructed to stand erect and return to the initial seated position as many times as possible within 30 seconds. Participants were monitored and instructed to fully sit between each stand. Only full standing positions were counted.

Maximal gait speed (GS) was assessed over a course of 10 m. Participants were asked to stand with their feet behind a starting line and, following a verbal cue, to start walking at their maximal safe walking pace. To reduce the effect of deceleration, participants were asked to continue beyond the 10 m. The time was measured with a stopwatch to the nearest 0.1 s. Maximal gait‐speed was calculated as the 10 m distance divided by time (m·s^−1^). High test–retest and inter‐rater reliability have previously been established for the physical tests mentioned earlier.^16^ More detailed information regarding the test‐protocols have been described elsewhere.^15^

### Circulating inflammatory markers

Blood samples were obtained from the antecubital vein of each participant on the day of clinical examination. Due to logistic circumstances blood samples were obtained in a non‐fasting state. Serum and plasma samples were collected in serum and EDTA‐treated tubes, respectively. Samples were centrifuged and distributed to polypropylene microcentrifuge tubes and stored at −80°C until analysed. Inflammatory biomarkers were measured in plasma samples using commercially available multiplex magnetic bead‐based immunoassay kits from Bio‐Rad (Bio‐Rad Laboratories, Inc.), as per the manufacturer's instructions. Multiplexed biomarkers were as follows: Interleukin (IL)‐1β, IL‐4, IL‐6, IL‐10, IL‐13, IL‐15, interferon‐γ (IFN)‐γ, and tumour necrosis factor (TNF)‐α. Cytokine levels were determined using the Bio‐Plex MAGPIX multiplex reader (Bio‐Rad Laboratories, Inc.) utilizing Luminex xMAP technology (Luminex), and data were analysed using the Bio‐Plex Manager Software, Version 6.1, Build 727. Two internal laboratory controls were included in all runs. Pooled plasma samples from healthy adults were split in two portions. The first portion comprised Control A while Control B consisted of the other portion spiked with the high standard from the Bio‐Rad immunoassay kit. Vortex and incubation periods, pipettes, and freezing/thawing cycles were consistent between assays to minimize inter‐assay variation. Standards, blanks, and controls were run in duplicate, while samples were run in a single well. Two blinded laboratory technicians performed all analyses. Lower limit of quantification (LLOQ) values and mean inter‐assay coefficients of variances (CVs) for the multiplexed cytokines can be found in *Supporting Information*, *Table* *S1*.

Serum levels of hsCRP were assessed using latex‐entrenched immune‐turbidimetry analyses (Roche/Hitachi automatic instrument COBAS®). The lower limit of detection (LLOD) was 0.15 mg/L and the inter‐assay CV was 2.1–8.4%. In the analyses of the first 200 samples, samples with extrapolated values under the LLOD (5.6%) were reanalysed using double sample volume. However, as these reruns only changed the concentration estimates by ±0.01, the subsequent extrapolated values were considered valid for the remaining samples.

### Statistics

Statistical analyses were performed in IBM® SPSS Statistics, version 25. Participants were divided into three groups representing young (20–39 years; 87 men and 119 women), middle‐aged (40–64 years; 219 men and 238 women), and elderly (65–93 years; 215 men and 282 women). Cytokine levels below the LLOQ were substituted by simple imputation of the value between 0 and the LLOQ based on the lowest accepted standard for the respective cytokine. IL‐10 and IL‐15 were excluded from all analyses as these inflammatory markers were virtually undetectable in the vast majority of participants.

Normality of distribution was assessed with Kolmogorov–Smirnov's test. Differences in clinical, functional, and body composition characteristics between groups were assessed using the following: independent‐samples Kruskal–Wallis tests, independent‐samples Mann–Whitney *U* tests, univariate analyses of variance, or independent *t* tests adjusted for multiple comparisons using Bonferroni's correction for each dependent variable. Biomarker profiles for the different age‐groups were presented as medians and interquartile range (IQR) with error bars displayed according to Tukey's method.

Sample size calculation for the association between inflammatory biomarker levels and muscle mass, visceral fat, muscle strength, and physical performance was based on previous results reported in a meta‐analysis (*r* = 0.20),^12^ *α* = 0.05 (two‐tailed), and *β* = 0.20.^17^ The required sample size was 194 participants. IL‐1β, IL‐4, and IL‐6 had a high (>50%) number of values below the LLOQ and were not included in the following analyses. Linear mixed‐effect models were used to explore the potential association between inflammatory biomarkers and relative ALM, visceral fat index, handgrip strength, STS performance, and maximal GS in elderly men and women, respectively. Sex was included as a fixed factor, participants' ID as a random factor, and the corresponding inflammatory biomarker level as a covariate. The maximum likelihood estimation and the best‐fitting covariance structure based on log‐likelihood values were considered for the model. The analyses were performed in steps resulting in three models representing (i) unadjusted analyses, (ii) analyses adjusted for age, and (iii) analyses adjusted for age and body mass index (BMI). Analyses with visceral fat index as the dependent variables were performed in steps resulting in two models representing (i) unadjusted analyses and (ii) analyses adjusted for age. Relative ALM, visceral fat index, muscle strength, and physical performance were used as continuous dependent variables in the analyses. Bonferroni's correction for multiple testing was applied to adjust for eight hypothesis tests (four inflammatory markers with separate analyses for men and women) for each dependent variable. Independent‐samples Mann–Whitney *U* tests were used to investigate whether elderly participants with low muscle strength, evaluated as low HGS, had higher levels of inflammatory markers compared with elderly with strength measures within the normal range. Low HGS was used as a categorical variable while inflammatory markers were used as continuous independent variables. All analyses were two‐sided with statistical significance set at *P* < 0.05.

## Results

### Descriptive characteristics of the study population

In total, data from 1160 men and women (mean age 58.5 years ± 17.4; 55% were female) were included. Demographic, anthropometric, and functional characteristics as well as body composition of the study population stratified by sex‐specific age‐groups are presented in *Table* 1. Relative to young men, middle‐aged and elderly men were significantly shorter, had a higher BMI, a larger accumulation of visceral fat, lower maximal GS, and lower STS (*P* < 0.05). Additionally, elderly men had significantly lower relative muscle mass (*P* < 0.05) and HGS (*P* < 0.05) compared with young men (*Table* 1). There was no difference in body mass between male age‐groups. Relative to young women, middle‐aged and elderly women were significantly heavier, had a higher BMI, a larger accumulation of visceral fat, lower HGS, lower maximal GS, and lower STS (*P* < 0.05) (*Table* 1). In addition, elderly women were significantly shorter compared with young women (*P* < 0.05). There was no difference in relative muscle mass between female age‐groups. Compared with women, men of all age‐groups were significantly taller, heavier, had a higher BMI, larger accumulation of visceral fat, higher relative muscle mass, higher HGS, and higher maximal gait‐speed (*P* < 0.05) (*Table* 1). Notably, there was no difference between young men and women regarding sit‐to‐stand performance, but middle‐aged and elderly men performed significantly better than their female counterparts (*P* < 0.05).

**Table 1:** Demographic, anthropometric and functional characteristics and body composition of the study population stratified by sex‐specific age‐groups

	Young men (*n* = 87)	Middle‐aged men (*n* = 219)	Elderly men (*n* = 215)	Young women (*n* = 119)	Middle‐aged women (*n* = 238)	Elderly women (*n* = 282)
Age (years)	31.3 ± 5.0	53.1 ± 7.1*	74.2 ± 6.0*	31.2 ± 5.0	52.9 ± 7.3*	75.2 ± 7.0*
Height (cm)	183.0 ± 6.7	180.7 ± 6.8*	176.9 ± 6.5*	168.2 ± 6.2^†^	167.3 ± 6.6^†^	162.7 ± 6.6*, ^†^
Body mass (kg)	83.1 ± 13.1	85.1 ± 12.3	82.8 ± 14.8	64.4 ± 10.2^†^	69.8 ± 12.1*, ^†^	67.7 ± 12.3*, ^†^
BMI (kg/m^2^)	24.8 ± 3.6	26.1 ± 3.7*	26.4 ± 4.2*	22.8 ± 3.4^†^	25.5 ± 4.5*, ^†^	25.6 ± 4.5*, ^†^
Visceral fat (g)^a^	424.3(243.3;688.0)	1187.1 (597.5;1965.3)*	1665.7 (1009.0;2419.4)*	132.2 (62.4;294.4)^†^	383.4 (211.0;885.2)*, ^†^	866.5 (487.5;1333.2)*, ^†^
Muscle (ALM/h^2^)	8.57 ± 1.04	8.42 ± 0.94	7.78 ± 0.97*	6.59 ± 0.77^†^	6.76 ± 0.83^†^	6.40 ± 0.86^†^
MVC, hand (kg)	53.0 ± 8.5	51.2 ± 8.3	40.3 ± 8.7*	34.4 ± 6.0^†^	32.0 ± 6.1*, ^†^	23.8 ± 5.7*, ^†^
30 s STS reps (*n*)	27.5 ± 5.5	23.3 ± 6.3*	16.4 ± 5.9*	26.9 ± 7.0	22.1 ± 6.5*, ^†^	15.0 ± 5.1*, ^†^
Gait speed (m/s)	2.80 ± 0.43	2.62 ± 0.43*	2.02 ± 0.51*	2.54 ± 0.39^†^	2.41 ± 0.52*, ^†^	1.78 ± 0.47*, ^†^

ALM, appendicular lean mass; BMI, body mass index; STS, sit‐to‐stand performance.

Results are presented as means ± SD unless otherwise indicated. Statistical significance set at *P* < 0.05. Results are Bonferroni corrected.

^a^ Result is presented as median (interquartile range).

* Denotes a significant difference from young sex‐specific group.

^†^ Denotes a significant difference from men.

### Low grade inflammation and the relation to age and sex

**Figure 1:**
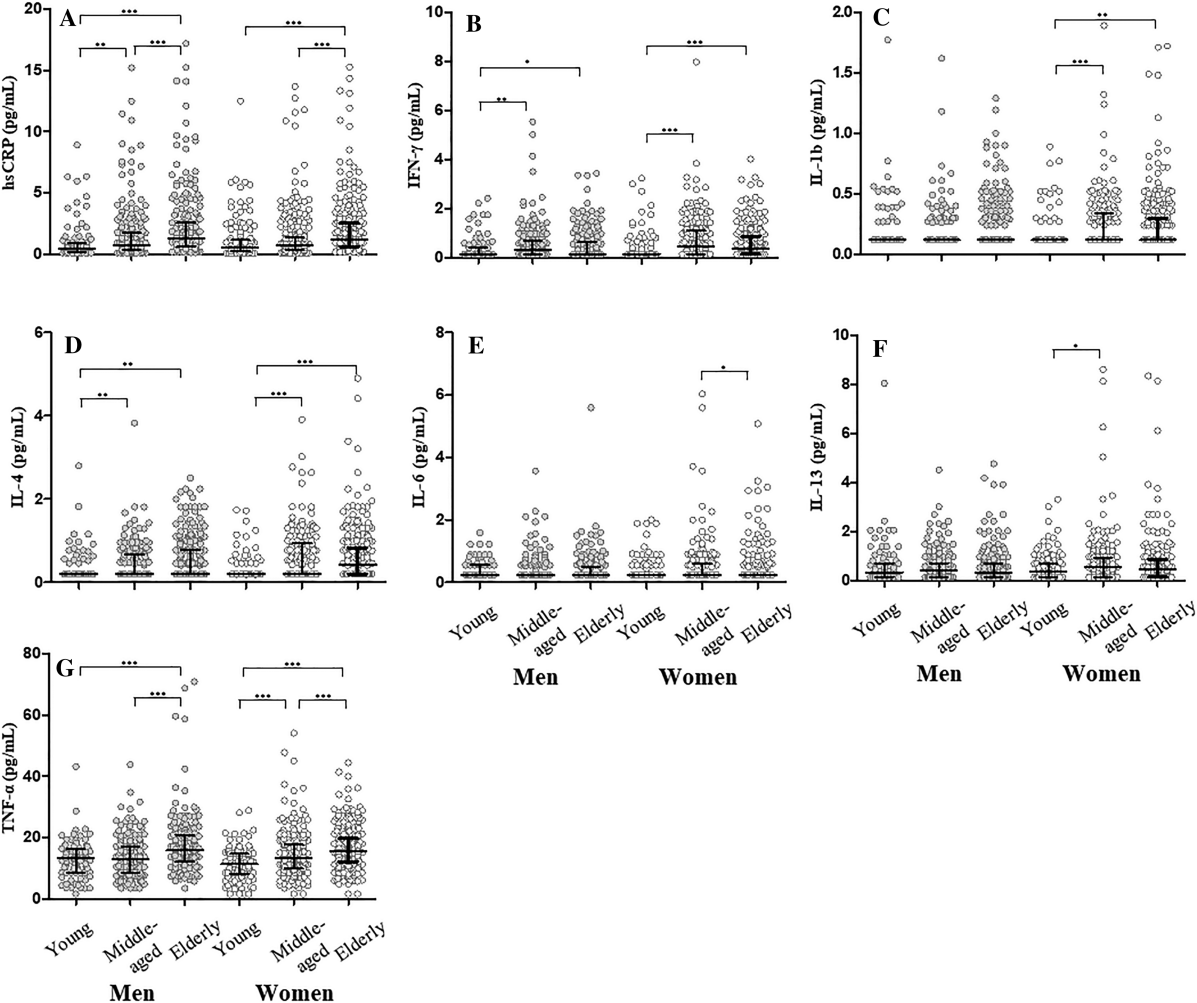
Biomarker profiles for seven inflammatory markers throughout the age‐span divided into young (22–39 years), middle‐aged (40–64 years), and elderly (65–93 years). *P < 0.05, **P < 0.01, ***P < 0.001. Results are Bonferroni corrected and presented as medians (interquartile range) with error bars displayed according to Tukey's method. Elderly men had significantly higher concentrations of hsCRP, TNF‐α, IL‐4, and IFN‐γ compared with young men, and significantly higher concentrations of hsCRP and TNF‐α compared with middle‐aged men. Elderly women had significantly higher concentrations of hsCRP, TNF‐α, IL‐1β, IL‐4, and IFN‐γ compared with young women, and significantly higher concentrations of hsCRP, TNF‐α, and IL‐6 compared with middle‐aged women.

Systemic levels of hsCRP, TNF‐α, IL‐6, IL‐1β, IL‐4, IL‐13, and IFN‐γ were obtained from all participants and included for further analyses in relation to age and sex. Inflammatory characteristics (median; interquartile range) stratified by sex‐specific age‐groups are presented in *Figure* 1. In 18 cases, participants were excluded due to outlying plasma or serum values as we assumed these values to reflect an acute inflammation rather than a chronic low‐grade inflammation. Outlier cut‐offs can be viewed in *Table* S2.

Relative to young men, elderly men had significantly higher concentrations of hsCRP (*P* < 0.01), TNF‐α (*P* < 0.001), IL‐4 (*P* < 0.01) and IFN‐γ (*P* < 0.05). In addition, elderly men had significantly higher concentrations of hsCRP (*P* < 0.001) and TNF‐α (*P* < 0.001) compared with middle‐aged men. There were no differences in IL‐1β, IL‐6, or IL‐13 concentrations across male age‐groups. Relative to young women, elderly women had significantly higher concentrations of hsCRP (*P* < 0.001), TNF‐α (*P* < 0.001), IL‐1β (*P* < 0.01), IL‐4 (*P* < 0.001), and IFN‐γ (*P* < 0.001). Furthermore, elderly women had significantly higher concentrations of hsCRP (*P* < 0.001), TNF‐α (*P* < 0.001), and IL‐6 (*P* < 0.05) compared with middle‐aged women.

Notably, there were no consistent sex‐differences in inflammatory biomarker concentrations. Young women had significantly lower TNF‐α (*P* < 0.05) concentrations compared to young men, whereas middle‐aged women had significantly higher concentrations of IL‐1β (*P* < 0.01), IL‐4 (*P* < 0.05), IL‐6 (*P* < 0.01), IL‐13 (*P* < 0.05), and IFN‐γ (*P* < 0.01) compared with middle‐aged men. Elderly women had significantly higher concentrations of IL‐4 (*P* < 0.05), IL‐13 (*P* < 0.05), and IFN‐γ (*P* < 0.05) compared with their male counterparts. There were no sex‐differences in hsCRP concentrations across age‐groups.

### Low‐grade inflammation in relation to body composition, muscle strength, and physical performance in elderly

Standardized beta coefficients with corresponding 95% confidence intervals (CIs) for the association of inflammatory markers with relative ALM (unadjusted, adjusted for age alone, and then for age and BMI) in elderly are shown in *Table* 2. After adjustment for age and BMI, inverse associations were observed between relative ALM and hsCRP in elderly men and women (*P* < 0.001), whereas no associations were observed between relative ALM and the remaining cytokines in neither men nor women. Further, inverse associations were observed between HGS and hsCRP in both elderly men (*P* < 0.01) and women (*P* < 0.05) (*Table* 3), which remained significant in women after adjusting for age and BMI (*P* < 0.05), but not in men (*P* = 0.056). No associations were observed between the remaining cytokines and HGS in neither elderly men nor women (*Table* 3).

**Table 2:** Association between relative appendicular lean mass (ALM/h^2^) and inflammatory markers in elderly men and women

	Unadjusted			Adjusted for age			Adjusted for age and BMI		
	Standardized β	95% CI	*P* value^a^	Standardized β	95% CI	*P* value^a^	Standardized β	95% CI	*P* value^a^
Men									
IL‐13 (pg/mL)	0.039	−0.095, 0.173	1.000	0.000	−0.125, 0.125	1.000	−0.005	−0.086, 0.076	1.000
IFN‐γ (pg/mL)	−0.017	−0.151, 0.118	1.000	−0.062	−0.188, 0.063	1.000	−0.045	−0.126, 0.036	1.000
TNF‐α (pg/mL)	−0.087	−0.221, 0.047	1.000	−0.014	−0.141, 0.113	1.000	−0.064	−0.145, 0.018	1.000
hsCRP (mg/L)	−0.007	−0.142, 0.127	1.000	0.080	−0.047, 0.207	1.000	**−0.171**	**−0.255, −0.087**	**<0.001**
Women									
IL‐13 (pg/mL)	−0.088	−0.205, 0.029	1.000	−0.073	−0.187, 0.042	1.000	−0.005	−0.073, 0.064	1.000
IFN‐γ (pg/mL)	−0.047	−0.164, 0.070	1.000	−0.068	−0.183, 0.047	1.000	−0.033	−0.101, 0.035	1.000
TNF‐α (pg/mL)	0.040	−0.078, 0.157	1.000	0.063	−0.052, 0.178	1.000	−0.028	−0.096, 0.041	1.000
hsCRP (mg/L)	0.141	0.025, 0.258	0.136	**0.161**	**0.047, 0.274**	**0.048**	**−0.126**	**−0.197, −0.056**	**0.008**

CI, confidence interval.

Bold values indicate *P* < 0.05.

^a^ Bonferroni corrected.

**Table 3:** Association between handgrip strength (kg) and inflammatory markers in elderly men and women

	Unadjusted			Adjusted for age			Adjusted for age and BMI		
	Standardized β	95% CI	*P* value^a^	Standardized β	95% CI	*P* value^a^	Standardized β	95% CI	P value^a^
Men									
IL‐13 (pg/mL)	0.026	−0.109, 0.160	1.000	−0.024	−0.143, 0.094	1.000	−0.025	−0.143, 0.093	1.000
IFN‐γ (pg/mL)	0.043	−0.091, 0.178	1.000	−0.014	−0.133, 0.105	1.000	−0.012	−0.130, 0.107	1.000
TNF‐α (pg/mL)	**−0.205**	**−0.337, −0.073**	**0.016**	−0.116	−0.236, 0.003	0.448	−0.123	−0.242, −0.004	0.344
hsCRP (mg/L)	**−0.229**	**−0.359, −0.098**	**0.008**	−0.129	−0.249, −0.009	0.280	−0.174	−0.299, −0.049	0.056
Women									
IL‐13 (pg/mL)	−0.038	−0.155, 0.079	1.000	−0.003	−0.106, 0.100	1.000	0.002	−0.101, 0.106	1.000
IFN‐γ (pg/mL)	0.090	−0.027, 0.207	1.000	0.045	−0.059, 0.148	1.000	0.047	−0.056, 0.151	1.000
TNF‐α (pg/mL)	−0.137	−0.253, −0.021	0.168	−0.086	−0.189, 0.017	0.824	−0.094	−0.198, 0.010	0.600
hsCRP (mg/L)	**−0.166**	**−0.282, −0.050**	**0.040**	−0.126	−0.229, −0.024	0.128	**−0.166**	**−0.275, −0.058**	**0.024**

CI, confidence interval.

Bold values indicate *P* < 0.05.

^a^ Bonferroni corrected.

Sit‐to‐stand performance was inversely associated with hsCRP in both elderly men (*P* < 0.001) and women (*P* < 0.05) but not after adjusting for both age and BMI (*Table* 4). Moreover, STS was positively associated with IL‐13 (*P* < 0.01) in elderly men but following adjustment for age and BMI it was no longer significant (*P* = 0.08). The remaining cytokines were not associated with STS in elderly men and women, respectively (*Table* 4). Following adjustment for age and BMI, maximal GS displayed an inverse association with hsCRP and a positive association with IFN‐γ in elderly women (*P* < 0.05, *Table* 5). No other associations were observed between inflammatory markers and GS (*Table* 5).

**Table 4:** Association between 30 s sit‐to‐stand performance and inflammatory markers in elderly men and women

	Unadjusted			Adjusted for age			Adjusted for age and BMI		
	Standardized β	95% CI	*P* value^a^	Standardized β	95% CI	*P* value^a^	Standardized β	95% CI	*P* value^a^
Men									
IL‐13 (pg/mL)	**0.195**	**0.063, 0.327**	**0.032**	0.155	0.033, 0.276	0.104	0.156	0.038, 0.274	0.080
IFN‐γ (pg/mL)	0.109	−0.024, 0.243	0.864	0.062	−0.062, 0.185	1.000	0.057	−0.064, 0.177	1.000
TNF‐α (pg/mL)	−0.164	−0.297, −0.032	0.120	−0.088	−0.213, 0.036	1.000	−0.074	−0.196, 0.047	1.000
hsCRP (mg/L)	**−0.295**	**−0.423, −0.166**	**<0.001**	**−0.215**	**−0.338, −0.093**	**0.008**	−0.165	−0.293, −0.038	0.088
Women									
IL‐13 (pg/mL)	−0.076	−0.193, 0.041	1.000	−0.048	−0.157, 0.060	1.000	−0.064	−0.171, 0.043	1.000
IFN‐γ (pg/mL)	0.146	0.030, 0.262	0.112	0.110	0.002, 0.218	0.368	0.103	−0.004, 0.209	0.472
TNF‐α (pg/mL)	−0.150	−0.266, −0.034	0.088	−0.110	−0.218, −0.001	0.376	−0.091	−0.198, 0.016	0.776
hsCRP (mg/L)	**−0.187**	**−0.303, −0.072**	**0.016**	**−0.156**	**−0.263, −0.049**	**0.040**	−0.109	−0.222, 0.004	0.472

CI, confidence interval.

Bold values indicate *P* < 0.05.

^a^ Bonferroni corrected.

**Table 5:** Association between maximal gait speed (m/s) and inflammatory markers in elderly men and women

	Unadjusted			Adjusted for age			Adjusted for age and BMI		
	Standardized β	95% CI	*P* value^a^	Standardized β	95% CI	*P* value^a^	Standardized β	95% CI	*P* value^a^
Men									
IL‐13 (pg/mL)	0.158	0.025, 0.291	0.160	0.108	−0.009, 0.225	0.552	0.109	−0.006, 0.225	0.504
IFN‐γ (pg/mL)	**0.192**	**0.060, 0.324**	**0.032**	0.136	0.019, 0.252	0.184	0.133	0.017, 0.248	0.192
TNF‐α (pg/mL)	−0.139	−0.272, −0.006	0.328	−0.045	−0.165, 0.074	1.000	−0.036	−0.154, 0.082	1.000
hsCRP (mg/L)	**−0.223**	**−0.354, −0.092**	**0.008**	−0.120	−0.239, −0.001	0.384	−0.084	−0.209, 0.041	1.000
Women									
IL‐13 (pg/mL)	0.037	−0.081, 0.154	1.000	0.080	−0.015, 0.175	0.800	0.071	−0.024, 0.165	1.000
IFN‐γ (pg/mL)	**0.204**	**0.089, 0.318**	**0.008**	**0.148**	**0.054, 0.242**	**0.016**	**0.144**	**0.051, 0.237**	**0.024**
TNF‐α (pg/mL)	−0.031	−0.148, 0.086	1.000	0.034	−0.062, 0.129	1.000	0.047	−0.049, 0.142	1.000
hsCRP (mg/L)	**−0.217**	**−0.332, −0.103**	**<0.001**	**−0.168**	**−0.262, −0.075**	**<0.001**	**−0.149**	**−0.248, −0.050**	**0.024**

CI, confidence interval.

Bold values indicate *P* < 0.05.

^a^ Bonferroni corrected.

Visceral fat accumulation normalized to height squared visceral fat index (kg/h^2^) was inversely associated with hsCRP in elderly men (*p* < 0.001) and women (p < 0.001) and remained associated after adjusting for age. Visceral fat index showed no associations with the remaining inflammatory markers in neither elderly men nor women (*Table* 6).

**Table 6:** Association between visceral fat index (VF/h^2^) and inflammatory markers in elderly men and women

	Unadjusted			Adjusted for age		
	Standardized β	95% CI	*P* value^a^	Standardized β	95% CI	*P* value^a^
Men						
IL‐13 (pg/mL)	−0.032	−0.152, 0.088	1.000	−0.036	−0.156, 0.085	1.000
IFN‐γ (pg/mL)	−0.086	−0.206, 0.033	1.000	−0.094	−0.215, 0.027	1.000
TNF‐α (pg/mL)	0.037	−0.083, 0.157	1.000	0.046	−0.076, 0.168	1.000
hsCRP (mg/L)	**0.306**	**0.193, 0.419**	**<0.001**	**0.327**	**0.212, 0.441**	**<0.001**
Women						
IL‐13 (pg/mL)	0.018	−0.087, 0.124	1.000	0.018	−0.088, 0.124	1.000
IFN‐γ (pg/mL)	−0.009	−0.115, 0.097	1.000	−0.008	−0.115, 0.098	1.000
TNF‐α (pg/mL)	0.098	−0.008, 0.203	0.552	0.098	−0.008, 0.203	0.552
hsCRP (mg/L)	**0.237**	**0.135, 0.340**	**<0.001**	**0.238**	**0.135, 0.340**	**<0.001**

CI, confidence interval.

Bold values indicate *P* < 0.05.

^a^ Bonferroni corrected.

Elderly men and women with low HGS (HGS below 36.11 kg for men and 20.17 kg for women (*n* = 145)^17^ had significantly higher levels of hsCRP (*P* < 0.01) and TNF‐α (*P* < 0.05) and were significantly older (73.3 ± 5.7 vs. 78.5 ± 7.1 years, *P* < 0.001) compared to elderly men and women with HGS within the normal range. There was no difference in remaining biomarkers, sex, or BMI between participants with low muscle strength and participants with strength measures within the normal range.

## Discussion

Chronic low‐grade inflammation has been studied in a wide range of populations and consistently suggested as one of the key factors involved in the development of sarcopenia.^11^, ^12^, ^13^, ^14^, ^18^, ^19^ However, it is still debatable whether chronic low‐grade inflammation is independently contributing to the age‐related changes in muscle mass and physical function or whether these markers merely reflect visceral or ectopic fat accumulation or an underlying chronic disease.^12^, ^20^ Several studies have investigated associations between inflammatory markers and muscle mass, muscle strength or physical function in elderly individuals with inconsistent results, depending on the population and inflammatory markers.^12^ In the present study, we have assessed biomarker profiles in a large healthy cohort and combined data with measurements of appendicular lean mass, visceral fat, HGS, STS, and maximal GS to explore potential associations between systemic inflammation and characteristics of age‐related sarcopenia (primary sarcopenia). Although we found significant increases in inflammatory biomarker concentrations with ageing, only higher hsCRP was weakly associated with having lower muscle mass, indicating that inflammation may not play a key role for the normal age‐related loss of muscle mass.

### Systemic low‐grade inflammation in relation to age and sex

As reflected in the inflammatory biomarker profiles for each sex‐specific age‐group (*Figure* 1), considerable variability was observed both within and between age‐groups emphasizing the heterogeneous nature of the complex inflammatory network. Nevertheless, we found a significant increase in inflammatory biomarker concentrations with ageing, confirming the presence of systemic low‐grade inflammation in both middle‐aged and elderly participants, in line with previous findings.^2^, ^4^, ^21^ The most robust increases were observed in systemic levels of hsCRP and TNF‐α, where elderly participants displayed significantly higher levels compared with both young and middle‐aged participants (*Figure* 1). This could, at least for hsCRP, be explained by an increase in visceral fat as visceral fat index was inversely associated with hsCRP in elderly men and women (*P* < 0.001), whereas visceral fat index showed no associations with the remaining inflammatory markers in neither elderly men nor women (*Table* 6).

Sex‐specific impacts of ageing on the immune system have previously been reported, with males showing a faster progression of immunosenescence compared to women.^22^ In the present cohort we observed higher levels of IL‐1β, IL‐4, IL‐6, IL‐13 and IFN‐γ in women compared to men within the middle‐aged and elderly age‐groups, however, overall sex differences were small and inconsistent across different inflammatory markers, and the contribution of sex to differences in biomarker concentrations is still unclear.

### Systemic low‐grade inflammation and sarcopenia

Chronic low‐grade inflammation and sarcopenia have independently been associated with negative health outcomes such as loss of independence and increased risk of morbidity, hospitalization, and mortality.^5^, ^6^ It is also evident that high cytokine levels in acute or chronic disease states regardless of age leads to muscle atrophy, accelerating the development of (secondary) sarcopenia.^12^, ^13^ Importantly, however, the role of chronic low‐grade inflammation for loss of muscle mass in otherwise healthy older individuals (primary sarcopenia) is less clear.^12^

Although higher hsCRP levels were significantly associated with having lower muscle mass in both elderly men and women (*Table* 2), these associations were somewhat weak (Std. *β* = −0.171 for men and 0.126 for women, *P* < 0.01). Notably, there were no associations between muscle mass and the remaining inflammatory markers in neither elderly men nor women, supporting the notion that chronic low‐grade inflammation may not play a key role for the normal age‐related loss of muscle mass in otherwise healthy individuals. Yet the present data revealed a relationship between inflammatory status and physical function, reflected by the inverse associations between high levels of hsCRP and low HGS (*Table* 3) in elderly women (*P* < 0.05) (*Table* 3). In the same cohort we have previously demonstrated that HGS declines much earlier in life compared with muscle mass.^15^ Thus, compared with young adults, HGS was significantly lower from the fifth decade whereas appendicular lean mass (ALM/h^2^) was significantly lower after the age of 70 years in men and 80 years in women.^15^ In addition to occurring earlier in life, the prevalence of low muscle strength was also much higher compared with low muscle mass. The fact that the decline in muscle strength far exceeds that of muscle mass during ageing indicates that the quality of the remaining muscle tissue is diminished with ageing. It is likely that an increase in intermuscular adipose tissue (IMAT) observed with ageing^20^, ^21^ is involved in this discrepancy. Besides the fact that IMAT may affect muscle architecture and consequently the force production of the muscle, it also releases a variety of inflammatory mediators resulting in both local^20^ and systemic inflammation.^23^ As such, future studies should investigate whether hsCRP may serve as a biomarker for detecting low muscle strength in otherwise healthy elderly, or whether hsCRP merely reflects an increase in visceral or ectopic fat accumulation.

We also found that higher levels of hsCRP were weakly associated with lower maximal GS in women (*P* < 0.05) (*Table* 5). In line with these findings, elevated levels of C‐reactive protein (CRP) and IL‐6 have been associated with lower muscle strength and poorer physical performance in 1020 men and women over the age of 65 from the InCHIANTI study,^24^ as well as in 542 older adults over the age of 55 across several health conditions.^19^ Somewhat to our surprise, we found a weak positive association between IFN‐γ and maximal GS in elderly women (*Table* 5). Although IFN‐γ is important for muscle regeneration following injury,^25^ it is also known to inhibit regeneration by suppressing M2 macrophage activation in models of persistent inflammation.^25^, ^26^ As such, the observed positive association between maximal GS and IFN‐γ seems counterintuitive and as these associations were weak, the clinical significance of this finding is questionable.

### Low functioning vs. high‐functioning elderly

It is evident that sarcopenia markedly increases the risk of disability, morbidity, hospitalization, and mortality.^5^, ^6^ In addition to understanding the aetiology of sarcopenia, it also seems important to discover sensitive predictors of early decline to prevent the development of sarcopenia. In an elegant study by Calvani and colleagues^21^ they used advanced statistical modelling to separate the profiles of low‐functioning and high‐functioning elderly according to a combination of systemic inflammation, physical function, and thigh composition.^21^ Importantly, high‐functioning elderly had inflammatory levels, thigh composition, and muscle strength parameters in‐between younger adults and more physically frail low‐functioning elderly, and approximately one third of the profiles of high‐functioning elderly were overlapping with the profiles of young adults, which was not caused by differences in age or comorbidity between low‐functioning and high‐functioning elderly,^21^ supporting the interplay between physical function, inflammatory status and body composition.

In the present study, participants were living independently and were generally healthy.^15^ In support of this, the prevalence of age‐related sarcopenia according to the EWGSOP guidelines^7^ was relatively small (3.6%).^15^ To further separate the group of elderly participants (*n* = 520) by functional status, they were divided into elderly participants with or without evident low muscle strength [HGS below 36.11 kg for men and 20.17 kg for women (*n* = 145)].^15^ Notably, these more physically frail elderly displayed a significant increase in hsCRP and TNF‐α, supporting an association between increased inflammation and lower muscle strength, especially in physically frail individuals. Of note, participants with low muscle strength were also significantly older compared to elderly with strength measures within the normal range (73.3 ± 5.7 vs. 78.5 ± 7.1 years, *P* < 0.001).

### Limitations

It is inevitable that the habitual level of physical activity plays an important role for physical function especially in old age. However, it was not possible to assess physical activity in the present study; thus, we are not able to establish the effect of physical activity on the measured variables. However, individuals with better physical function are likely to have higher levels of physical activity. Moreover, blood‐samples were obtained in a non‐fasting state as not all subjects could be tested in the morning. The inflammatory markers are pleiotropic proteins known to act in complex and dynamic networks with numerous feedback mechanisms and their effect depends on the presence of other inhibiting, modulating or synergistic cytokines or soluble receptors. Hence, the inflammatory biomarkers included in the present study may not be sufficient to elucidate the potential relationship between low‐grade inflammation and loss of muscle mass, strength, and physical function with ageing. Of note, the investigation of multiple outcomes increases the risk of Type I errors. Lastly, the vast majority of participants were Caucasians, which limits the generalizability to other ethnic groups.

## Conclusion

In conclusion, our results demonstrate that inflammatory markers increase with age, with the most robust increases seen regarding hsCRP and TNF‐α, which were also especially elevated in more physically frail elderly supporting the association between low‐grade systemic inflammation and physical function. In contrast, only high levels of hsCRP were weakly associated with low muscle mass and positively associated with visceral fat and low physical function, suggesting that chronic low‐grade inflammation may not play a key role in the age‐related loss of muscle mass. Future studies should investigate whether hsCRP may serve as a sensitive biomarker for detecting low muscle strength in otherwise healthy elderly, or whether hsCRP merely reflects an increase in visceral or ectopic fat accumulation.

## Funding

The work is supported by funding from the Novo Nordisk Foundation; grant number NNF18OC0052826.

## Conflicts of interest

None.

## Supporting information


**Table S1.** LLOQ and mean CV% of cytokines included in the multiplex assay.

**Table S2.** Cut‐offs used to define outliers.*
