# Supplementary material for: Associations between inflammatory markers, body composition, and physical function: the Copenhagen Sarcopenia Study
Source: J Cachexia Sarcopenia Muscle. 2021 Oct 27;12(6):1641–52. doi: 10.1002/jcsm.12832 (PMC8718077; doi:10.1002/jcsm.12832)
Supplement: Supplementary file 1 — Table S1. LLOQ and mean CV% of cytokines included in the multiplex assay. [file JCSM-12-1641-s002.pdf]

Associations between inflammatory markers, body composition, and physical function: The Copenhagen Sarcopenia Study

Kamper R.S., Alcazar J., Andersen L.L., Haddock B., Jørgensen N.R., Hovind P., Suetta C.

The Journal of Cachexia, Sarcopenia and Muscle

Corresponding author

Rikke Stefan Kamper

Email: rikke.stefan.kamper.01@regionh.dk

Telephone: +4520446333

Geriatric Research Unit, Department of Geriatric and Palliative Medicine,

Bispebjerg and Frederiksberg Hospital,

University of Copenhagen,

Denmark

SUPPORTING INFORMATION

The lower limit of quantification (LLOQ) for each biomarker included in the multiplex assay was based on the expected concentration of the lowest standard following an assessment of the recovery-range of the lowest standard. A recovery range between 60-140% was deemed acceptable. Cytokine levels below the LLOQ were substituted by simple imputation of the value between 0 and the LLOQ based on the lowest accepted standard for the respective cytokine. LLOQ values and mean inter-assay coefficients of variances (CV) for the multiplexed cytokines are displayed in Table S1.

Table S1. LLOQ and mean CV% of cytokines included in the multiplex assay

	LLOQ (pg/ml)	Mean inter-assay CV%
IL-1β	0.24	15%
IL-4	0.40	21%
IL-6	0.48	12%
IL-10	6.48	14%
IL-13	0.28	19%
IL-15	33.68	18%
IFN-γ	0.28	40%
TNF-α	3.24	20%
