# Supplementary material for: Associations between inflammatory markers, body composition, and physical function: the Copenhagen Sarcopenia Study
Source: J Cachexia Sarcopenia Muscle. 2021 Oct 27;12(6):1641–52. doi: 10.1002/jcsm.12832 (PMC8718077; doi:10.1002/jcsm.12832)
Supplement: Supplementary file 2 — Table S2. Cut‐offs used to define outliers.* [file JCSM-12-1641-s001.pdf]

Associations between inflammatory markers, body composition, and physical function: The Copenhagen Sarcopenia Study

Kamper R.S., Alcazar J., Andersen L.L., Haddock B., Jørgensen N.R., Hovind P., Suetta C.

The Journal of Cachexia, Sarcopenia and Muscle

Corresponding author

Rikke Stefan Kamper

Email: rikke.stefan.kamper.01@regionh.dk

Telephone: +4520446333

Geriatric Research Unit, Department of Geriatric and Palliative Medicine,

Bispebjerg and Frederiksberg Hospital,

University of Copenhagen,

Denmark

SUPPORTING INFORMATION

Following assessment of the biomarker profile for young subjects (20-39 years), outliers were defined as being more than 4 times higher than the 97.5 percentile of the young group. In 18 cases, participants were excluded due to outlying plasma or serum values; as we assumed these values to reflect an acute inflammation rather than a chronic low-grade inflammation. Outlier cut-offs are displayed in Table S2.

*Table S1. Cut-offs used to define outliers**

	Cut-off (number of participants)
IL-1β	3.04 pg/ml (6 participants)
IL-4	5.48 pg/ml (2 participants)
IL-6	6.27 pg/ml (5 participants)
IL-13	8.97 pg/ml (5 participants)
IFN-γ	8.72 pg/ml (3 participants)
TNF-α	90.63 pg/ml (2 participants)
hsCRP	25.32 mg/l (8 participants)

**The same participant may appear multiple times*
